# Giving feedback on others’ writing

**DOI:** 10.1007/s40037-018-0492-z

**Published:** 2019-01-07

**Authors:** Chris Watling, Lorelei Lingard

**Affiliations:** Centre for Education Research and Innovation, Schulich School of Medicine and Dentistry, Western University, London, Ontario Canada

In the Writer’s Craft section we offer simple tips to improve your writing in one of three areas: Energy, Clarity and Persuasiveness. Each entry focuses on a key writing feature or strategy, illustrates how it commonly goes wrong, teaches the grammatical underpinnings necessary to understand it and offers suggestions to wield it effectively. We encourage readers to share comments on or suggestions for this section on Twitter, using the hashtag: #how’syourwriting?

Recently, a medical educator colleague of ours did a remarkable thing. In an effort to illuminate coaching practices for fellow medical teachers, he called upon his experience as a musician, staging a live cello master class and featuring himself as the learner. He’s already a fine musician, but his coach wanted him to bring some emotional depth to his playing—a quality in the performances of great musicians that we might be inclined to think of as intangible, even unteachable. But she did not simply tell him to play with more emotion. Instead, she talked about the pressure on the bow, the arc of the bow stroke, the movement of his body, the position of the bow on the strings, and the way he handled tempo and dynamics. She deconstructed ‘emotion’ into its component technical parts, and our colleague’s playing transformed in front of our eyes.

While this master class was about music, it mirrored the challenge and the joy of giving others feedback on their writing. Most of us not only write, but also read, edit, critique, and support the writing of students or colleagues. Handled poorly, feedback on writing can be confusing, unhelpful, and even discouraging. But handled well, feedback on writing—like feedback on cello playing—has the power to transform.

Ideally, feedback should feel like a conversation between reader and writer. To achieve this, it helps to establish what the conversation is going to be about. Writers should make focused requests of their readers, such as ‘I’d like to know if my Problem/Gap/Hook is clear in the opening paragraph’ or ‘I’m wondering if my paragraph transitions are working’ or ‘I’m concerned the discussion just repeats the results but I’m not sure how to fix it’. When writers don’t make specific requests, readers should ask for direction: ‘What would you like me to focus on? Logic of the argument? Sentence and paragraph construction? Success with achieving a particular tone?’ If writer and reader are not sure what to focus on, consider attending first to story, then to structure, and finally to style as a way of organizing the feedback [1].

Even with focused requests for feedback, readers will almost certainly notice other aspects of the writing that need strengthening. Remember, though, writers can only absorb so much feedback at once. Maintain your focus on a few areas and just note other issues to be addressed later. For instance, if you see that a writer struggles to use commas appropriately, flag one or two in the draft and add a comment box that says you will address this grammar issue later. Or, if the tone of the writing is too casual for a research manuscript, mention that this is something to discuss a few drafts further along. Put a pin in the issue, without overwhelming the writer with too much feedback at once.

Agreeing on a feedback focus is necessary but not sufficient for success. In addition, your actual comments on the writing need to be specific in order to be actionable. This can be quite tricky. Many readers possess good instincts for when something is wrong with the writing and know how to fix it themselves. But many also lack the vocabulary and knowledge to *name* the problem and *explain* the fix. Lacking this, readers default to offering generic comments (‘awkward’, ‘unclear’ and ‘vague’ are favourites) or fixing the problem without commenting at all. Neither approach helps the writer to diagnose and solve their recurring writing weaknesses.

What is the reader who is not a writing expert to do? First, name the problems you can. For instance, instead of scribbling ‘confusing’ beside a paragraph, remind the writer about paragraph structure: ‘A paragraph needs an opening topic sentence to orient the reader to its main idea, and that idea should develop as the paragraph unfolds. I think either your topic sentence is missing, or it’s the wrong topic sentence for this paragraph, or the paragraph just includes too many ideas’. Second, when your instinct tells you something isn’t working but you can’t say why, just say so. For instance, many writers create long, meandering sentences that lose the reader, but you might not know exactly what’s gone grammatically wrong. In such cases, share your reading experience: ‘This very long sentence lost me about here. You could try breaking it into 2 or 3 sentences or using stronger punctuation to help the reader follow the relationships between these ideas’.

Comments like these remind us that giving feedback on someone’s work and editing someone’s work are two different tasks. When offering feedback, try to resist the urge to simply rewrite. Think about making comments for consideration, rather than making changes that the writer can simply accept. Rampant use of the ‘track changes’ function in Word may reduce the likelihood of the writer really engaging with the feedback. Sometimes all a writer is looking for is some careful copy editing. But if they’ve asked for feedback, provide it in a way that they can really engage with the process. If a writer’s paragraph is unconvincing, for example, try something like: ‘This key paragraph isn’t as compelling as it could be. The problem may be that you have used a lot of ‘to be’ verbs. Try replacing a few of them with stronger, action-oriented verbs to better command the reader’s attention’. There’s a much greater chance the writer will be able to use your feedback in other situations if you highlight concerns, offer possible diagnoses, articulate an option or two for improvement, and provide a rationale for your suggestions.

Although mere rewriting can be dispiriting, a demonstration of how your feedback can be put to work can be powerful. Therefore, aim for a balance of telling and showing in your feedback. If you use a rewrite to show how a passage may be strengthened, couple your edits with an explanatory note. For example, because parallel structure is an effective device for strengthening the impact of writing, you might wordsmith a paragraph to inject a dose of parallelism. Nothing wrong with that—but the rewrite is much more effective as feedback if accompanied by a comment that explains ‘I’ve tried to create a parallel structure here by matching the grammatical construction of the first three sentences; I think this change makes the ideas more persuasive’.

Remember that writing—even academic writing—can be deeply personal. Feedback on writing, therefore, is a delicate business; a critique of writing as product can easily be misinterpreted as a critique of writer as person. When feedback threatens self-esteem or stirs strong emotions, it becomes very challenging for individuals to process and integrate, even if it is accurate and potentially useful [2, 3]. Advice about feedback typically encourages us to focus on the task rather than on the individual in order to defuse threats to self-esteem and mitigate negative emotion [4]. But because the task of writing is not emotionally neutral, this feat may be easier said than done. Acknowledging this challenge honestly may be helpful. Focusing on the experience of the reader also helps; for example, instead of saying ‘Your use of jargon is confusing’, consider instead ‘I’m worried that some readers may not understand these terms—perhaps a definition would help here’.

A little praise doesn’t hurt either. Look for strengths and successes to point out to the writer, like ‘what a powerful verb!’, ‘lovely turn of phrase here’, ‘strong transitions between these paragraphs’, or ‘nice job balancing a formal research tone with more conversational moments’. Such feedback reinforces good practice and bolsters confidence, and we all need our writing confidence bolstered! When you are working closely with a writer and seeing multiple drafts, you have the opportunity to comment specifically on improvements from past drafts. This signals to the writer that her efforts to change were worth it, that she is gaining expertise, and that pleasing you is not a random event. We say the last only partly tongue-in-cheek, because we know that writers may perceive contradictions in multiple rounds of feedback on their writing. Sometimes, as the drafts evolve, we readers change our minds. This is okay; in fact it can be very instructive for writers if the reasoning is explicit. After all, successful writing is a craft, not a recipe. In this spirit, we have found ourselves writing comments that admit, ‘I know I suggested to try this new organization, but I don’t think it’s working. The logic seems to fall apart. I propose we go back to the earlier structure but put more emphasis on explicit signposting to make it easier for readers to follow’.

Writing feedback is a powerful tool. Use it consciously, and with care. At the end of our colleague’s cello master class, one of the audience members asked the teacher about her philosophy when giving feedback to musicians. She thought for a moment, then said ‘You must be sure that you don’t kill the joy’. Writing, like playing music, can be technical and frustrating. But it can also be joyous, and supporting writers to find that joy may be the key to sustaining their engagement in the challenging craft of writing.
